# New Family Members of FG Repeat Proteins and Their Unexplored Roles During Phase Separation

**DOI:** 10.3389/fcell.2021.708702

**Published:** 2021-07-12

**Authors:** Yoichi Shinkai, Masahiro Kuramochi, Takamitsu Miyafusa

**Affiliations:** ^1^Molecular Neurobiology Research Group, Biomedical Research Institute, National Institute of Advanced Industrial Science and Technology (AIST), Tsukuba, Japan; ^2^Graduate School of Science and Engineering, Ibaraki University, Hitachi, Japan; ^3^Bio-System Research Group, Bioproduction Research Institute, National Institute of Advanced Industrial Science and Technology (AIST), Tsukuba, Japan

**Keywords:** FG repeat, phase separation, intrinsically disordered protein, nuclear pore, P granule, keratohyalin granule

## Abstract

The condensation and compartmentalization of biomacromolecules in the cell are driven by the process of phase separation. The main effectors of phase separation are intrinsically disordered proteins, which include proteins with a phenylalanine-glycine (FG) repeat domain. Our understanding of the biological function of FG repeat proteins during phase separation has been mainly derived from recent research on a member of the nuclear pore complex proteins, nucleoporins containing FG repeat domain (FG-NUPs). FG-NUPs form meshwork structures by inter- and intra-molecular FG domain interactions, which confine the nucleo-cytoplasmic exchange. Whereas FG-NUPs localize in the nuclear membrane, other FG repeat proteins reside in the cytoplasm and the nucleoplasm, and the biological function of the FG repeat domain of these proteins is not well described. In the present review, we list the FG repeat proteins that are known to phase separate in the cell, and review their biological functions. We extract the unraveled features of FG repeat proteins as an activator of barrier formation and homotypic cell-cell interactions. Understanding the regulatory mechanisms of FG repeat proteins will provide a potential delivery tool for therapeutic reagents.

## Introduction

The phenomenon of phase separation in living organisms was first observed in the P granules of *Caenorhabditis elegans* (Brangwynne et al., 2009). Since then, liquid-liquid phase separation has been reported as a driving force for the formation of various membraneless organelles, such as nucleoli, stress granules, and microtubule organizing centers, leading to the reconsideration of various intracellular phenomena from the viewpoint of phase separation (Brangwynne et al., 2011; Molliex et al., 2015; Rale et al., 2018). Organelles with membranes, such as mitochondria and the endoplasmic reticulum, carry out efficient reactions via a series of internal functions by assembling proteins. Organelles without membranes, which are formed by phase separation, are also thought to play a role in assembling proteins related to continuous biological reactions into liquid-like “condensates,” where the concentration of proteins rise by about 100-fold in the micromolar or millimolar range (Li et al., 2012; Kanaan et al., 2020; Yoshizawa et al., 2020). Phase separation also plays an important role in controlling gene expression by partitioning the genome into heterochromatin and euchromatin regions (Larson et al., 2017; Strom et al., 2017; Sabari et al., 2018; Gibson et al., 2019). Thus, phase separation is involved in intracellular compartmentalization and enrichment, which provides the basis for the proper functioning of cells.

The condensates formed by phase separation contain multiple proteins and RNA. Many of the proteins that constitute this condensate have intrinsically disordered regions (IDRs) that drive phase separation through a variety of transient and multivalent interactions (Krainer et al., 2021). IDRs are sometimes referred to as low-complexity (LC) regions because they lack a fixed three-dimensional structure, and are generally composed of a limited number of amino acids. Highly dynamic liquid-like condensate matures over time and transitions to gel-like or solid-like condensates (Lin et al., 2015; Patel et al., 2015). The components that exist inside phase-separated organelles are generally classified as scaffolds and clients. While the scaffold protein is essential for phase separation, the client interacts with the scaffold and gathers inside the condensate, but is not essential for phase separation itself (Banani et al., 2016). Dozens of proteins are present inside phase-separated organelles such as stress granules, and the inside of the phase-separated condensate is in an extremely heterogeneous state (Markmiller et al., 2018; Youn et al., 2018). The proteins that can access the inside are dependent on the scaffold of the condensate.

**FIGURE 1:**
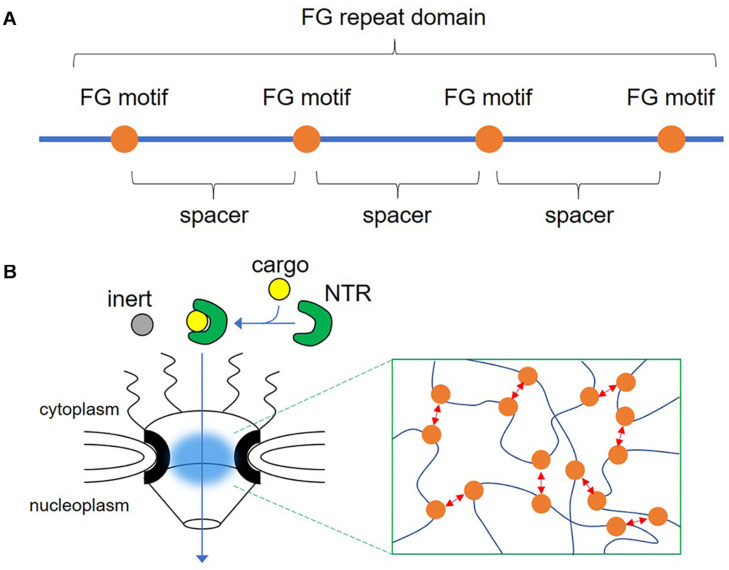
Illustration of the phenylalanine-glycine (FG) repeat domain and the nuclear pore complex. **(A)** The FG repeat domain is composed of consecutive repeats of the FG motif (orange circle) and spacers, which lack a consensus sequence but are rich in serine and threonine residues. **(B)** Drawing of the nuclear pore complex indicating the transport of a nuclear transport receptor (NTR) and its cargo through the central channel composed of nucleoporins containing FG repeat domains (blue cloud). Inset shows dynamic multivalent interaction between FG motifs (red arrows).

The role of phenylalanine-glycine (FG) repeat proteins as scaffold proteins has been investigated in nuclear pores. The hallmark of FG repeat proteins is that FG motifs are separated by spacers that lack a consensus sequence, but are rich in serine and threonine residues (Figure 1A). Recently, remarkable progress has been made in phase separation research, and the phase separation of various intrinsically disordered proteins and their intracellular functions have been clarified. Despite the existence of the FG repeat domain in such proteins, its role remains largely unexamined. In the present review, we discuss the role of the FG repeat domain in proteins other than FG-nucleoporins (FG-NUPs), and propose new roles for them in phase separation.

## The Function of FG Repeat Domains in NPCs

Nuclear pore complexes (NPCs) located in the nuclear pores are conduits that connect the nucleoplasm and cytoplasm, which are separated by the nuclear envelope. A single NPC can mediate the translocation of approximately 1,000 molecules per second in both directions (Ribbeck and Gorlich, 2001). In addition to these high-speed material exchanges, another important role of NPCs is the selective permeability barrier. Small molecules of 30 kDa or less can be permeated by passive diffusion, but larger molecules are more difficult to pass through, depending on their molecular weight (Timney et al., 2016). For these large molecules to pass through, they must be carried by nuclear transport receptors (NTRs). By ensuring such rapid substance transport and maintaining the selective permeability barrier, the nucleus localizes specific biopolymers inside and performs important compartmentalization for efficient functional expression. The mechanisms by which these seemingly contradictory NPC properties of rapid mass transport and selective permeation are achieved will be discussed in detail below.

NPCs are composed of approximately 30 nucleoporins and large protein complexes ranging from ∼60 MDa in *Saccharomyces cerevisiae* to ∼130 MDa in vertebrates (Reichelt et al., 1990; Rout et al., 2000; Denning et al., 2003). Of these, nucleoporins localized in the central channel of NPCs are called FG-NUPs because they have a consecutive repeat of the FG motif. This FG repeat domain is an IDR without a structure (Denning et al., 2003; Denning and Rexach, 2007), with the denatured region protruding toward the inside of the pore (Ribbeck and Gorlich, 2001; Chug et al., 2015; Kosinski et al., 2016). One NPC contains multiple copies of more than 10 FG-NUPs, and a large number of FG repeat domains fill the central plug of the nuclear pores (Kim et al., 2018). The key to solving the mystery of why NPCs have multiple FG repeat domains was *in vitro* reconstitution experiments of the nuclear pores, which revealed that the FG repeat domain isolated from yeast Nsp1 forms a hydrogel that recapitulated the permeability barrier of NPCs (Frey et al., 2006; Frey and Gorlich, 2007). Low molecular weight proteins and NTRs could pass through this hydrogel, but proteins with molecular weights above 30 kDa could not. These characteristics are commonly reported in other FG-NUPs (Labokha et al., 2013; Schmidt and Görlich, 2015). The copy number of NUPs per NPC has been quantified (Rout et al., 2000; Kim et al., 2018), and the local concentration of the FG motifs in the central plug of the nuclear pore *in vivo* is estimated to be 50 mM (Bayliss et al., 1999; Frey and Gorlich, 2007). Because of this, a large number of FG repeat domains are gelled, even in the nuclear pores of live cells. Electron tomography of the hydrogels of FG repeat domains from FG-NUPs revealed that FG repeat domains formed interlaced amyloid fibers that created a meshwork structure inside the hydrogels (Milles et al., 2013). Consistently, structural analyses of NPCs using high-speed atomic force microscopy and cryo-electron tomography showed that FG-NUPs extend filamentous protrusions into the central channel (Eibauer et al., 2015; Mohamed et al., 2017). Interestingly, despite the intrinsically disordered nature of the FG repeat domain, it can constitute a structured meshwork and provide permeability (Figure 1B).

FG repeat domains of FG-NUPs are phase-separated *in vitro* (Schmidt and Görlich, 2015). This indicates that the FG repeat domains have the ability to autonomously aggregate to form NPC pores. The driving force of this autonomous aggregation is derived from phenylalanine residues, because the phenylalanine to serine mutation in the FG repeat domain of yeast Nsp1 impairs hydrogel formation (Frey et al., 2006; Frey and Gorlich, 2007). On the other hand, strong binding was maintained with a phenylalanine to tyrosine mutation, suggesting that the aromatic ring is important, because hydrophobic and/or aromatic (π-π) interactions between phenyl groups form highly elastic hydrogels from the FG repeat domain (Frey and Gorlich, 2007). The FG motif in the FG domain interacts intra-and inter-molecularly (Patel et al., 2007; Krishnan et al., 2008). In addition, aliphatic alcohols, which inhibit hydrophobic interactions, reversibly increase permeability to macromolecules that cannot pass through the nuclear pores (Ribbeck and Gorlich, 2002). The hydrophobic interactions between phenylalanine residues therefore effectively function as a barrier in nuclear pores. However, the FG domain is not only composed of phenylalanine and glycine, and its properties differ depending on the amino acid composition of the spacers between FG motifs (Ader et al., 2010). For example, the asparagine-rich (N-rich) FG domain on the N-terminal side of Nsp1 tends to form hydrogels, but the C-terminal FG domain, which contains several charged amino acids, does not form hydrogels. The β-sheet interactions commonly found in asparagine and glutamine-rich (NQ-rich) amyloids in N-rich regions also contribute to the formation of hydrogels. In experiments with Thioflavin T (ThT), NQ-rich Nup100 (28%) and Nup116 (26%) condensates were reported to be ThT-positive (Schmidt and Görlich, 2015). In contrast, the *in vitro* condensates formed by the FG domain from Nup98 extracted from various organisms have the ability to phase-separate and pass only NTRs, despite their completely different NQ ratios and ThT staining patterns, suggesting that these ratios and staining patterns alone cannot explain the modes of interaction in the FG repeat domain (Schmidt and Görlich, 2015). The inclusion of a large amount of hydrophobic amino acids could lead to strong cohesion between the FG repeat domains.

## Non-Biased Search for Novel FG Repeat Proteins

The FG repeat domain is characterized by a simple amino acid sequence but with distinct physicochemical properties. We aimed to discover novel FG repeat proteins, which might have essential roles in biological function other than forming nuclear pore. Tools for the prediction of IDRs based on amino acid sequences are available online, but their predictive accuracy is not always adequate (Nielsen and Mulder, 2019). Therefore, it may be more practical to focus on the frequency of FG dipeptides for predicting the FG domain. It should be noted that both phenylalanine and glycine are rich in transmembrane helices of membrane proteins (Baker et al., 2017) and consequently that FG dipeptides are also often observed in membrane proteins, such as the photosystem II CP43 reaction center protein from *Thermosynechococcus elongatus* (Young et al., 2016).

The FG domain of human Nup98, one of the most well-analyzed nuclear envelope proteins, contains 38 FG dipeptides out of approximately 500 amino acids on the N-terminal side. Schmidt and Görlich (2015) extensively searched the database to identify 666 Nup98 homologous protein candidates. These protein groups contained 43 ± 6 FG dipeptides in the FG domain of 549 ± 87 amino acids. These values are important benchmarks for the size and density of the FG domain.

We referred to known nuclear envelope proteins to determine the minimum sequence length and number of dipeptides accumulated to define a functional FG domain. *Saccharomyces cerevisiae* contains Nup98 paralog proteins, Nup145, Nup100, and Nup116. Of these three proteins, Nup145 has the smallest FG domain, containing 11 FG dipeptides in approximately 200 amino acids (Wente and Blobel, 1994). Nup1 of *S. cerevisiae* carries an FG domain containing five FG dipeptides in approximately 200 amino acids near the C-terminus (Davis and Fink, 1990). In the case of NUP62 from *Homo sapiens*, approximately 150 amino acids near the N-terminus contain five FG dipeptides (Clarkson et al., 1996). Based on these examples, we hypothesized that the FG domain requires at least five FG dipeptides in a region of 200 amino acids. Among the 563,552 sequences from UniProtKB/Swiss-Prot, a protein database containing reviewed, manually annotated entries (UniProt, 2019), 2,863 proteins met our criteria. Representative entries from *C. elegans* and humans are listed in Tables 1, 2, respectively.

**TABLE 1:** The top 15 phenylalanine-glycine (FG)-rich proteins in *C. elegans*.

Gene name	Maximum number of FG motifs in 200 amino acids	Total number of FG motifs	Total length (aa)	Association with phase separation	Description
K01A6.4	21	23	284	?	Uncharacterized
glh-2	20	35	974	Yes (Spike et al., 2008)	DEAD-box helicase in P granules (Spike et al., 2008; Sheth et al., 2010)
glh-4	20	46	1,156	Yes (Spike et al., 2008)	DEAD-box helicase in P granules (Spike et al., 2008; Sheth et al., 2010)
pqn-75	20	20	539	?	Prion-like Q/N-rich protein involved in stress resistance and thermotolerance (Rochester et al., 2017)
npp-10	17	37	1,678	Yes (Voronina and Seydoux, 2010)	Nuclear pore complex protein
glh-1	16	21	763	Yes (Updike et al., 2011)	DEAD-box helicase in P granules (Spike et al., 2008; Sheth et al., 2010)
npp-11	15	31	805	?	Nuclear pore complex protein
npp-7	14	31	1,217	Yes (Voronina and Seydoux, 2010)	Nuclear pore complex protein
ego-2	12	22	1,494	?	Bro1-domain protein involved in germline proliferation (Liu and Maine, 2007)
npp-4	12	16	538	Yes (Updike et al., 2011)	Nuclear pore complex protein
nlp-14	12	12	224	?	NLP family neuropeptide
rde-12	11	16	959	Yes (Shirayama et al., 2014)	DEAD-box helicase in P granules (Spike et al., 2008; Sheth et al., 2010)
npp-14	11	20	1,390	?	Nuclear pore complex protein
ddx-19	11	21	1,022	Yes (Sheth et al., 2010)	DEAD-box helicase in P granules (Spike et al., 2008; Sheth et al., 2010)
npp-1	11	13	639	?	Nuclear pore complex protein

**? Indicates unknown association with phase separation, aa, amino acids.**

**TABLE 2:** The top 15 phenylalanine-glycine (FG)-rich proteins in humans.

Gene name	Maximum number of FG motif in 200 amino acids	Total number of FG motifs	Total length (aa)	Association with phase separation	Description
NUP214	19	44	2,090	?	Nuclear pore complex protein
NUP98	19	41	1,817	Yes (Schmidt and Görlich, 2015)	Nuclear pore complex protein
TRO	17	45	1,431	?	Trophinin
NUP153	14	29	1,475	?	Nuclear pore complex protein
POM121	14	24	1,249	?	Nuclear pore complex protein
POM121B	14	24	834	?	Nuclear pore complex protein
POM121C	14	24	1,229	?	Nuclear pore complex protein
FLG2	13	41	2,391	Yes (Quiroz et al., 2020)	Flaggrin-2, expressed in the epidermis (Wu et al., 2009)
SBSN	12	16	590	?	Suprabasin, expressed in the epidermis (Aoshima et al., 2019)
KRT3	12	15	628	?	Type II Keratin
KRT2	11	13	639	?	Type II Keratin, expressed in the epidermis (Collin et al., 1992)
KRT10	11	12	584	Yes (Quiroz et al., 2020)	Type I Keratin, expressed in the epidermis (Kartasova et al., 1993)
NUP42	10	12	423	?	Nuclear pore complex protein
KRT76	10	11	638	?	Type II Keratin
KRT1	10	10	644	?	Type II Keratin, expressed in the epidermis (Kartasova et al., 1993)

**? Indicates unknown association with phase separation, aa, amino acids.**

## FG Repeat Proteins in P Granules

**FIGURE 2:**
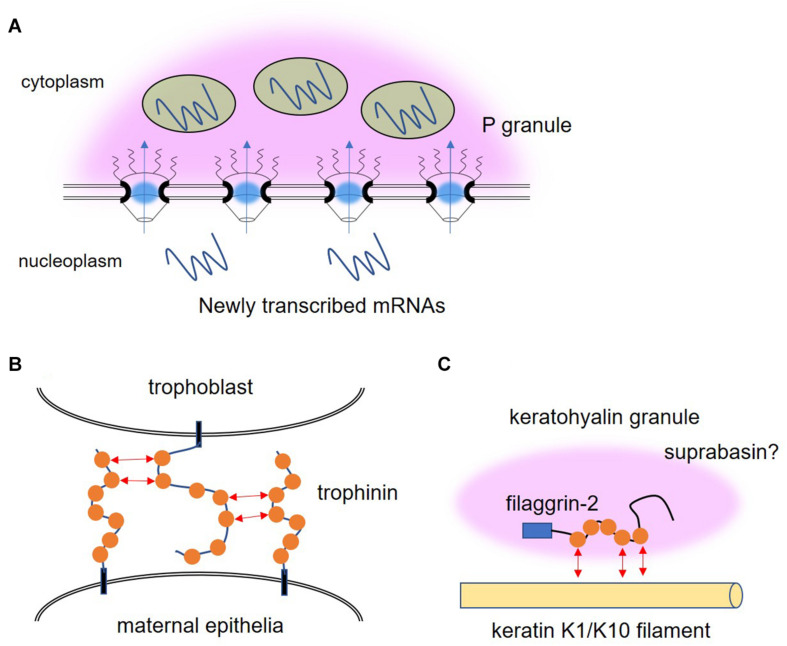
Illustration of the possible roles of phenylalanine-glycine (FG) repeat proteins other than in the nuclear pore. **(A)** P granule (pink cloud) is attached to the nuclear pores in the adult germline of *C. elegans* by the interaction between FG repeats in GLH proteins, the main constitutive components of P granules, and the meshwork structure formed by nucleoporins containing FG repeat domains. Newly transcribed mRNAs traverse P granule and the small RNA machinery (green circles) that resides in P granules might prevent excessive flux of RNA to the cytoplasm. **(B)** The FG motifs (orange circle) of trophinin mediate homophilic adhesion between trophoblast and maternal epithelia during embryonic implantation. **(C)** FG repeat-containing proteins, filaggrin-2, keratin K1, keratin K10, and suprabasin, are expressed in the same epidermal keratinocytes and contribute to the formation of keratohyalin granules, which drive skin barrier formation.

P granules are germline granules in *C. elegans* that are found exclusively in germ cells and prevent somatic differentiation to maintain totipotency (Seydoux, 2018). In addition to FG-NUPs, there are proteins carrying the FG repeat domain in P granules, namely DEAD-box helicase proteins GLH-1 (germ line helicase-1), GLH-2 (germ line helicase-2), GLH-4 (germ line helicase-4), RDE-12 (RNAi defective-12), and DDX-19 (DEAD box helicase homolog-19) (Spike et al., 2008; Sheth et al., 2010; Shirayama et al., 2014). GLH proteins are the main constitutive components of P granules, although ectopic expression of GLH-1 was not sufficient to form perinuclear granules unless PGL-1 (P granule abnormality-1), an RNA-binding protein with an arginine-glycine-rich RGG box, was expressed simultaneously. As shown in Table 1, the FG repeat number of these proteins was comparable to that of FG-NUPs in *C. elegans*. Although P granules float asymmetrically in the cytoplasm of one-cell embryos, P granules are attached to the nuclear pores in the adult germline (Figure 2A). An intriguing hypothesis was proposed to explain this phenomenon in which P granules are anchored to the nuclear pore by the interaction between FG repeats in GLH proteins and the meshwork structure formed by FG-NUPs (Updike et al., 2011). Consistent with this model, the deletion of the FG repeat domain from GLH-1 impaired the perinuclear localization of P granules (Chen et al., 2020). Similarly, CeNup98, an FG-NUP in *C. elegans*, is required for the anchoring of P granules at the nuclear pore (Voronina and Seydoux, 2010). These observations suggest that the interplay between FG domains of GLH proteins and FG-NUPs underlies the perinuclear localization of P granules. P granules dissolved after exposure to 1,6-hexandiol, which inhibits hydrophobic protein-protein interactions, suggesting that the hydrophobic interactions of FG-repeat domains could also be a driving force of P granule phase separation (Updike et al., 2011).

It is thought that P granules contain FG repeats to further regulate the selectivity of transport through the nuclear pore that they are connected to. P granules are not required for embryonic development, as embryos lacking P granule assembly can develop into larval worms (Gallo et al., 2010). On the other hand, P granules are essential for germ cell differentiation. RNAi knockdown of PGL-1, PGL-3, GLH-1, and GLH-4 simultaneously leads to the complete loss of P granules, and germ cells aberrantly differentiate into somatic cells and display a neuronal cell structure (Updike et al., 2014). Newly transcribed mRNAs are exported through P granule-associated nuclear pore complexes, and sequester the mRNAs in P granules (Sheth et al., 2010). Dicer, a riboendonuclease in the small RNA pathway, interacts with GLH-1 (Beshore et al., 2011). Furthermore, several argonaute proteins are localized in the P granules (Campbell and Updike, 2015; Aoki et al., 2021). Thus, the small RNA machinery that resides in P granules might prevent the excessive flux of RNA that is involved in aberrant differentiation and suppress gene expression in the cytoplasm.

## FG Repeat Proteins in Extracellular Region

Human trophinin is also an FG repeat protein expressed in germ cells (Fukuda et al., 1995). Trophinin is the intrinsic membrane protein expressed in both trophoblast and maternal epithelia and is involved in embryonic implantation through a unique homophilic adhesion mechanism at their respective apical cell membranes (Fukuda et al., 1995; Suzuki et al., 1999). The FG repeat domain located in the extracellular region of trophinin might be responsible for this intercellular adhesion via interaction between FG motifs (Figure 2B). Bystin is a cytoplasmic protein that intermediates the formation of the trophinin-bystin-tastin complex by directly binding both trophinin and tastin, and also functions as a sensor for homophilic adhesion (Suzuki et al., 1998). When trophinin-mediated cell adhesion occurs, bystin is released from trophinin, allowing the activation of epidermal growth factor receptor B4 (ErbB4) protein kinase, resulting in the promotion of invasion and proliferation (Fukuda and Sugihara, 2007; Sugihara et al., 2007). Interestingly, direct binding between the trophinins of neighboring cells might cause the release of bystin from trophinin, raising the hypothesis that the interaction between extracellular intrinsically disordered FG-rich domains induces structural changes in its cytoplasmic region to release bystin. Applying this hypothesis to the phase separation that occurs in the cytoplasm would mean that the homotypic interactions of LC domains can cause conformational changes in the remaining ordered domains of the protein, which can themselves trigger signaling. Since other adhesion molecules, such as integrins, selectins and cadherins, have also been reported as essential molecules in the process of implantation (Kimber and Spanswick, 2000; Singh and Aplin, 2009), we assume that several types of interaction are employed for the strict spatiotemporal control of each step in the cascade of implantation. Trophinin is involved not only in embryonic implantation but also in the invasive potential of cancer (Chen et al., 2007; Harada et al., 2007; Chang et al., 2009). A characteristic feature of phase separation driven by FG motifs, such as those found in FG-NUPs in the nuclear pore, is that they can produce interactions that are multivalent and transient yet collectively strong. As discussed in the next chapter on the relationship between NTR and FG-NUPs, these properties may provide a solution to the constraint of balancing association and dissociation during cell invasion, where cells must migrate while generating strong cell-cell interactions.

## NTR Is a Chaperone That Controls Phase Separation of Macromolecular Condensates

**FIGURE 3:**
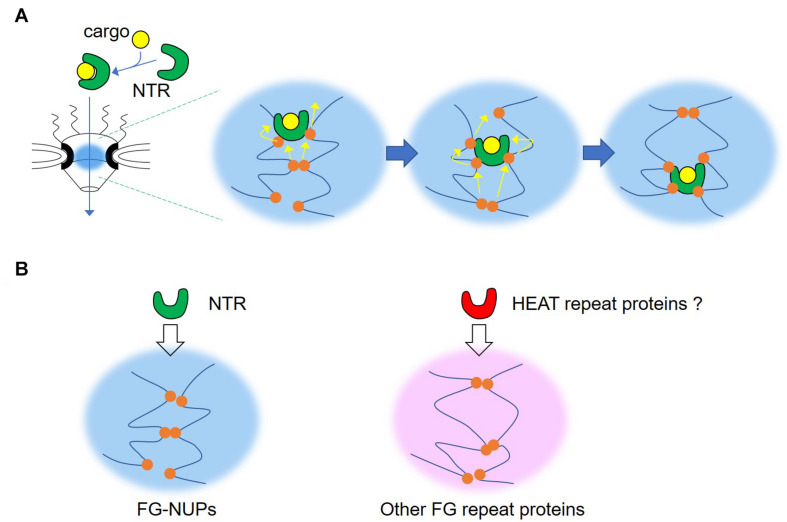
Illustration of the mode of permeation of the nuclear pore of nuclear transport receptor. **(A)** Phenylalanine-glycine (FG) repeats interact with multiple binding sites on the nuclear transport receptor (NTR) surface, both multivalently and transiently. FG motifs can be displaced by other competing FG motifs, and slide on the surface of NTRs. By repeating this process, the NTR gradually moves to the nucleoplasmic side. **(B)** The NTR is responsible for specific transport through nucleoporins containing FG repeat domains (FG-NUPs). A similar mechanism might be shared among other FG repeat proteins and HEAT repeat proteins.

The selective gating function of NPCs is dependent on the permeability of FG repeats, and also on the orchestration of FG repeats and NTRs. Large cargoes are usually unable to pass through the nuclear pore but can do so efficiently when they are bound to NTRs. According to *in vitro* measurements, FG-NUPs and NTRs bind too tightly to explain the rapid transport of cargo through NPCs by NTRs since high binding affinity suggests long residence times when NTRs cross barriers rich in FG-NUPs (Pyhtila and Rexach, 2003; Frey and Gorlich, 2009; Tetenbaum-Novatt et al., 2012). On the other hand, a low binding affinity may blunt the specificity of nuclear transport of cargo by NTRs. Nuclear magnetic resonance (NMR) and molecular dynamics simulations have revealed that FG repeats are highly dynamic and remain disordered upon binding to NTRs without any conformational changes (Hough et al., 2015; Milles et al., 2015). FG repeats multivalently interact with multiple binding sites on the NTR surface with a low affinity. FG motifs can be displaced by other competing FG motifs and slide on the surface of NTRs (Raveh et al., 2016). These features ensure ultrafast binding and unbinding between FG-NUPs and NTRs, leading to the fast passage of NTRs through FG-NUPs in NPCs (Figure 3A). The surface properties of these NTRs are directly related to the surface amino acid composition, which strongly affects transport efficiency. NTR-like green fluorescent protein (GFP), a GFP redesigned to have a hydrophobic surface and lysine to arginine substitution, exceeds the transport rate of NTRs, while the original GFP before modification had a transport rate that was over 100 times lower (Frey et al., 2018). Multiple NTR molecules are required to transport large cargo molecules through the FG-NUP barrier (Tu et al., 2013; Schmidt and Görlich, 2015). The exposed hydrophobic and arginine residues promote NPC passage through hydrophobic interactions with FG repeats and cation-π interactions with the phenyl group of FG motifs.

Recently, NTRs have been intensively studied as regulators of phase separation. Karyopherin β2 (Kapβ2), a non-classical NTR, also known as transportin 1 or importin β2, inhibits the phase separation of RNA-binding protein fused in sarcoma (FUS), which leads to the collapse of FUS condensates (Yoshizawa et al., 2018). Although Kapβ2 strongly interacts with the proline-tyrosine nuclear localization signal (PY-NLS) of FUS, Kapβ2 also inhibits the phase separation of FUS without PY-NLS (Gonzalez et al., 2021). The C-terminal RGG region of FUS binds directly to Kapβ2 and is necessary and sufficient for the phase separation of FUS (Dormann et al., 2012; Gobl et al., 2016; Hofweber et al., 2018). Therefore, phase separation occurs because of the interaction between the RGG regions of each FUS, and Kapβ2 inhibits this interaction. On the other hand, in the case of full-length FUS, cation-π interactions between C-terminal arginine residues and N-terminal tyrosine residues drive phase separation, which is regulated by Kapβ2 (Qamar et al., 2018). Furthermore, Kapβ2 inhibits the fibrillization of FUS, TAF15, EWSR1, hnRNPA1, and hnRNPA2 (Guo et al., 2018). Likewise, karyopherin β1 (Kapβ1), also called importin beta, can inhibit FUS condensates (Yoshizawa et al., 2018). The phase separation of FG repeat domains from the FG-NUP NUP98A was partially disrupted by Kapβ1 (Schmidt and Görlich, 2015). Under *in vitro* molecular crowding conditions, FG repeat domains isolated from FG-NUPs can aggregate and form elongated amyloid fibrils, which are also inhibited by Kapβ1 (Milles et al., 2013). Thus, karyopherin family members may generally have the ability to control the phase separation of macromolecules. The majority of karyopherin proteins are occupied by multiple repeats of HEAT motifs, which are found in a number of cytoplasmic proteins and derived from the acronym of those four proteins (Huntingtin, Elongation factor 3, protein phosphatase 2A, and TOR1); for a review of HEAT domains, see Yoshimura and Hirano (2016). The flexible structural property of the HEAT repeat upon binding with FG motifs also contributes to the effective transport of NTRs through the nuclear pore (Yoshimura et al., 2014). So far, the best available hypothesis is that proteins that have hydrophobic and arginine-rich surfaces and structural flexibility dependent on multiple HEAT repeats can efficiently pass through the nuclear pores and control phase separation. It may be interesting to search for proteins with HEAT repeats corresponding to FG repeat proteins proposed in the present review (Figure 3B); for example, PAA-1, *the C. elegans* homolog of PR65, is one of the HEAT repeat proteins localized in the P granule.

## FG Repeats for a Skin Barrier Formation

The proteins with the most FG motifs per 200 amino acids included filaggrin-2, keratin 1 (K1), keratin 2 (K2), keratin 10 (K10), and suprabasin (Table 2). Interestingly, these proteins are expressed in the same epidermal keratinocytes (Figure 2C), which proliferate in the basal layer of the skin, move up and mature through the spinous and granular layers, and terminally differentiate in the cornified layer (Collin et al., 1992; Kartasova et al., 1993; Wu et al., 2009; Aoshima et al., 2019). In particular, filaggrin is involved in the barrier function of the skin through phase separation (Quiroz et al., 2020). Keratohyalin granules are formed when keratinocytes reach the granular layer. The main constituent of these keratohyalin granules is filaggrin, which forms droplets. After granule formation, the keratohyalin granules are captured by keratin fibers and become firmly immobile, so that the fusion of droplets is inhibited. Filaggrin-2, like filaggrin, is present in keratohyalin granules and is degraded by proteases during final differentiation (Wu et al., 2009). Filaggrin and filaggrin-2 have an S100 domain at the N-terminal region, which dimerizes and promotes phase separation (Quiroz et al., 2020). The FG-rich region is included in the A-type repeats of filaggrin-2. Although the precise role of the FG-rich A-type repeats is unknown, A-type repeats are incorporated in cornified envelopes that act as a permeability barrier (Alberola et al., 2019). In a 3-dimensional-reconstructed human epidermis (RHE) model, the knockdown of filaggrin-2 caused abnormalities in keratinocyte differentiation, resulting in abnormal skin barrier function (Pendaries et al., 2015). In addition, non-sense mutations in filaggrin-2 are associated with dermatitis in African American patients (Margolis et al., 2014).

Multiple FG motifs are present in the N-terminal LC domain of K1 and K10, which copolymerize in pairs and form filaments. Keratinocytes express terminal differentiation-specific keratin K1 and K10 during differentiation, and the pre-existing keratin K5/K14 network is replaced by K1/K10 (Kartasova et al., 1993). Although keratin K5/K14 assembles into 10-nm filaments that tend not to interact with each other, K1/K10 filaments aggregate markedly *in vitro* (Eichner et al., 1986). These differences in characteristics are due to the sequence and nature of the LC domain of keratin proteins. Interestingly, the number of FG motifs in the LC domain of K1/K10 is much higher than that of K5/K14. The LC domain of K1/K10 is thought to protrude along the surface of filaments and plays a role in both filament-filament and filament-other protein interactions (Fuchs, 1998). Keratin filaments specifically interact with filaggrin, which is known as a keratin filament-aggregating protein (Steinert et al., 1981). In fact, when mCherry was fused with the K10 LC domain and co-expressed with filaggrin, it was incorporated into filaggrin droplets (Quiroz et al., 2020). Furthermore, in HaCaT immortalized human keratinocytes, in the presence of K10 expression, the critical concentration required for phase separation of filaggrin was reduced. Therefore, the interaction with K10 promotes the phase separation of filaggrin. This interaction between filaggrin droplets and keratin fibers is reminiscent of the relationship between NPCs and P granules. The switch of expression from K4/K15 to K1/K10 during terminal differentiation may be a mechanism for ensuring interaction and phase separation with filaggrins.

Suprabasin is also expressed in the differentiating epidermal cells above the granular layer (Aoshima et al., 2019). In an RHE model, deficiency in suprabasin caused immature keratohyalin granules but did not affect the other markers of epidermal differentiation. Suprabasin-null mice showed skin barrier dysfunction as embryos but not after birth, and ultrastructural abnormalities in the stratum corneum and immature keratohyalin granules were observed (Nakazawa et al., 2020). Furthermore, the amount of suprabasin was significantly decreased in atopic dermatitis patients (Sakabe et al., 2014; Li et al., 2018). These reports provide evidence to support the function of suprabasin in skin barrier formation. However, the role of the FG repeat domain is completely unknown since suprabasin possesses a putative secretory signal peptide at its N-terminal (Matsui et al., 2004). Changes in suprabasin expression in various diseases such as cancer and autoimmune disorders suggest that suprabasin has diverse functions (Pribyl et al., 2021).

All these FG repeat-containing proteins contribute to the formation of keratohyalin granules, which promote the destruction of cellular organelles as an essential feature of skin barrier formation (Quiroz et al., 2020). Considering the function of FG repeats in NPCs, it is intriguing to imagine that a network composed of keratins and keratohyalin granules forms a barrier against the external environment as well as a selective gating barrier. Further studies will uncover why these proteins expressed in the epidermis commonly have FG repeats.

## Conclusion and Perspectives

Previous studies have clarified the role of FG repeats in nuclear pores. However, FG repeats also exist in other proteins where their roles have not yet been clarified. In this review, we examined other proteins with FG repeats and hypothesized that they have unraveled functions as an activator of barrier formation and homophilic adhesion. In particular, proteins with FG repeats are densely expressed in the skin and could play an important role in maintaining the skin barrier. Given the role of NTR in crossing FG-rich nuclear pores, substances that mimic NTR may be able to easily cross the skin barrier and may provide new clues for transdermal drug transfer. Of course, this role has not yet been experimentally verified, and other FG repeat proteins may have a completely different role because the condensates of FG repeat proteins must be composed of a considerable concentration of FG motifs to have a barrier function, which is shown by the lack of barrier function in hydrogels with low concentrations of FG motifs (Frey and Gorlich, 2007; Milles et al., 2013). According to our definition, many human proteins contain FG repeats, and proteins with FG repeats have already been investigated for their function in other contexts. Therefore, it is anticipated that research on these proteins from the viewpoint of FG repeats will advance our understanding of entirely new roles for these proteins. Analytical tools using microfluidic devices (Celetti et al., 2020), imaging systems with high spatiotemporal resolution (Mohamed et al., 2020), and proteomics tools that combine these are expected to play an important role in such research.

## Author Contributions

YS conceived and designed the draft. TM proposed the definition of FG repeat proteins. MK extracted FG repeat proteins from UniProt database. YS, MK, and TM wrote and edited the manuscript. All authors contributed to the article and approved the submitted version.

## Conflict of Interest

The authors declare that the research was conducted in the absence of any commercial or financial relationships that could be construed as a potential conflict of interest.
